# Effects of dietary metabolizable energy level on hepatic lipid metabolism and cecal microbiota in aged laying hens

**DOI:** 10.1016/j.psj.2024.103855

**Published:** 2024-05-15

**Authors:** Anjian Li, Hong Hu, Ying Huang, Fuyan Yang, Qianhui Mi, Liqiang Jin, Hongli Liu, Qiang Zhang, Hongbin Pan

**Affiliations:** ⁎ Yunnan Provincial Key Laboratory of Animal Nutrition and Feed Science, Faculty of Animal Science and Technology, Yunnan Agricultural University, Kunming, 650201, China; † WOD Poultry Research Institute, Beijing, 100193, China

**Keywords:** late laying hen, dietary metabolizable energy (ME), hepatic lipid metabolism, cecal microbiota, gut health

## Abstract

Lipid metabolic capacity, feed utilization, and the diversity of gut microbiota are reduced in the late laying stage for laying hens. This experiment aimed to investigate the effects of different levels of dietary metabolizable energy (**ME**) on hepatic lipid metabolism and cecal microbiota in late laying hens. The 216 Peking Pink laying hens (57-wk-old) were randomly assigned to experimental diets of 11.56 (HM = high ME), 11.14 (MM = medium ME), or 10.72 (LM = low ME) MJ of ME/kg, with each dietary treatment containing 6 replicates per group and 12 chickens per replicate. The HM group showed higher triglyceride (**TG**), total cholesterol (**T-CHO**), and low-density lipoprotein cholesterol (**LDL-C**) concentrations in the liver compared with the LM group; second, the HM group showed higher TG concentration and the LM group showed lower T-CHO concentration compared with MM group; finally, the HM group showed a lower hepatic lipase (**HL**) activity compared with the MM and LM groups (*P* < 0.05). There was a significant difference in the microbial community structure of the cecum between the HM and MM groups (*P* < 0.05). The decrease of dietary ME level resulted in a gradual decrease relative abundance of Proteobacteria. At the genus level, beneficial bacteria were significantly enriched in the LM group compared to the MM group, including *Faecalibacterium, Lactobacillus*, and *Bifidobacterium*, (linear discriminant analysis [LDA] >2, *P* <0.05). In addition, at the species level, *Lactobacillus crispatus, Parabacteroides gordonii, Blautia caecimuris*, and *Lactobacillus johnsonii* were significantly enriched in the LM group (LDA>2, *P* < 0.05). The HM group had a higher abundance of *Sutterella spp*. compared to the LM group (LDA>2, *P* <0.05). In conclusion, this research suggests that the reduction in dietary energy level did not adversely affect glycolipid metabolism or low dietary ME (10.72 MJ/kg). The findings can be helpful for maintaining intestinal homeostasis and increasing benefit for gut microbiota in late laying hens.

## INTRODUCTION

Energy content is a key nutritional component of the diet of laying hens (Musigwa et al., 2021). All life activities of poultry, from growth and development, production and reproduction, to subtle physiological and biochemical reactions, are closely linked to dietary energy (Bain et al.,2016). Appropriate levels of ME in the diet are necessary to promote healthy growth in poultry (Chang et al.,2023). Dietary energy affects growth performance, reproductive capacity, and production costs of poultry (Peng et al.,2023). Energy feed, led by corn and supplemented with grease, is an important source of energy for poultry and is a major component of poultry feeds (Selaledi et al., 2020). A diet with insufficient dietary energy can slow down the growth rate of poultry and reduce production performance, while excessive energy can affect the metabolism of laying hens, reduce product quality, and lead to a decrease in economic benefits of laying hens’ production (Gao et al., 2021). Therefore, dietary energy intake is important for laying hens. The liver and gut are essential for nutrient metabolism and overall health in laying hens (Jian et al., 2021). However, their capacity declines with age, affecting production performance and causing economic losses.

The liver plays a central role in energy metabolism and is the main organ involved in biotransformation and detoxification in the laying hens (Qi et al., 2019). The metabolic burden on the liver can be increased by excess energy intake, and impaired lipid metabolism in the liver can affect intestinal health (Ringseis et al., 2020). Excessive energy in the diet leads to fat deposition and decreased carcass quality (Miao et al. 2017), while the prevalence of fatty liver has been shown to increases with age (Gu et al., 2021). These findings indicate that the lipid metabolic capacity of laying hens is easily affected at the aging stage, and suggest that more attention should be paid to the health status of aged laying hens.

The gut microbiota of poultry is influenced by diet, age, antibiotic use, and infection by pathogenic organisms (Zimmermann et al., 2019). In poultry, nutrient digestion and absorption primarily occur in the small intestine (including the duodenum, jejunum, and ileum) (Wen et al., 2021). The cecum, a major component of the large intestine, can produce volatile fatty acids through microbial fermentation to provide energy for host activities (Binek et al., 2017). Fermentation by cecal microorganisms helps increase the productivity of poultry and resist invasion by pathogenic bacteria (Bjerrum et al., 2006). The composition of cecal microorganisms is affected by changes in dietary energy levels (Daniel et al., 2014). It has been recently reported that appropriate energy levels help to enhance breast muscle composition, improve meat quality and nutritional value, and improve the gut microbiota of native growing hens (Chang et al. 2023).

The liver energy metabolism capacity and gut microbiota diversity decline during the aged laying period, these declines affect production performance and cause substantial economic losses (Gan et al. 2020). Therefore, the regulation of dietary metabolizable energy (**ME**) levels is particularly important for laying hens entering the aged stages. However, whether dietary ME levels affect liver lipid metabolism ability and gut microbiota of laying hens in the aged laying period remains unclear. This study aimed to analyze the effects of dietary ME on the glycolipid metabolic capacity and gut microbiota in aged laying hens, to provide a theoretical basis for healthy rearing of laying hens at the aged stage of egg production and for the rational use of feed resources.

## MATERIALS AND METHODS

### Experimental Design and Chickens

Ethical Statement: All animal studies were approved by the Ethics Committee of Yunnan Agricultural University (Approval Number: 202202001).

A total of 216 Peking Pink laying hens (57-wk-old) with similar body weight (1.9 kg) and initial laying rates (83%) were supplied by YunLingGuangDaYukou Poultry Co., Ltd, Yunnan, China. Hens were randomly allocated to 3 groups with 6 replicates per group and 12 chickens per replicate (1 replicate in 3 cages, 4 birds per cage). This study used a single factor experimental design. The chickens were fed with 3 levels of dietary metabolizable energy (**ME**): Low ME group (10.72 MJ/kg; LM), medium ME group (11.14 MJ/kg; MM), and high ME group (11.56 MJ/kg; HM), respectively. The ME levels were adjusted according to the recommended levels for Peking Pink laying hens (NY / T33-2004). The composition and nutrient levels of the diets are shown in Table 1, which met all NRC (1994) and “ChickenFeeding Standard” (NY/T 33−2004) requirements. Each hen was provided with the same corn-soybean meal basal diet of approximately 112 g per day and had free access to water, based on the management procedure for breeding Peking Pink laying hens (Yukou Poultry Co., Ltd, Beijing, China). The trial consisted of a 1-wk adaptation period and 10-wk formal testing cycle. Throughout the entire trial, the average ambient temperature and relative humidity were maintained at 18 to 25°C and 40 to 60%, respectively.

**Table 1:** Composition and chemical analysis of the basic diets (air-dried basis, %).

Item	LM	MM	HM
Ingredient, %			
Corn	59.06	61.60	59.06
Soybean meal	23.69	24.70	25.20
Wheat bran	6.17	2.00	2.00
Calcium hydrogen phosphate	0.67	0.80	0.82
Soybean oil	0.00	0.60	2.64
limestone powder	9.41	9.30	9.28
Premix 1	1.00	1.00	1.00
total	100.00	100.00	100.00
Nutrition level 2			
ME, MJ/kg	10.72	11.14	11.56
crude protein, %	16.29	16.29	16.29
Crude fiber, %	2.74	2.57	2.56
Ca, %	3.67	3.67	3.67
Total P, %	0.52	0.52	0.52
Effective P, %	0.27	0.27	0.27
Lay, %	0.83	0.85	0.85
Met+Cys, %	0.55	0.55	0.55

1 Supplying per kilogram of diet:VA 8,000-10,000 IU, VD_3_ 2,200-5,000 IU, VE 13 IU, VK_3_ 1.4-4.8 mg, VB_1_ 1.8 mg, VB_2_ 3.0 mg, VB_6_ 2.0 mg, VB_12_ 0.01 mg, nicotinamide 20 mg, D- calcium pantothenate 10 mg, folic acid 0.55 mg, D- biotin 0.15 mg, choline 380 mg, Fe 60 mg, Cu 8 mg, Mn 60 mg, Zn 60 mg, I 0.35 mg, Se 0.12-0.48 mg, Ca 60-180 mg.

2 The nutrient level is the calculated value.

### Sample Collection

At the end of the experimental period, one laying hen per replicate was randomly selected for slaughter. The content of the cecum was collected in a frozen storage tube and rapidly stored in a liquid nitrogen tank for analysis of cecal microbiota. The liver was immediately placed in a self-sealing bag and stored in a -20°C refrigerator for determination of relevant biochemical indicators.

### Determination of Liver Lipid Indicators and Methods

Prior to the determination of lipid accumulation in the liver, approximately 1 g of liver sample was homogenized with a pre-cooled saline solution or anhydrous ethanol at a ratio of 1:9 (wt/vol) using a glass tissue homogenizer. The supernatant, obtained through centrifugation at 12,000 rpm at 4°C for 10 min. The concentration of total protein (BCA, A045-3-2), triglycerides (TG, A110-2-1), total cholesterol (T-CHO, A111-2-1), and low-density lipoprotein cholesterol (LDL-C, A113-2-1), as well as the activities of hepatic lipase (HL, A067-1-2), lipoprotein lipase (LPL, A067-1-2), and lipase (LPS, A054-1-1) were measured using commercial kits obtained from the Nanjing Jiancheng Institute of Bioengineering (Jiangsu, China). In addition, high-density lipoprotein cholesterol (HDL-C, HDL-C-1-G) was determined using a commercial kit (Keming Biotechnology Research Institute, Suzhou, China).

The kits were applied as per the recommendations of the manufacturer. Finally, the results were normalized against the total protein concentration in the supernatant.

### Sequencing of Microbiota From Cecal Contents Samples and Data Analysis

Total genomic DNA was extracted from all cecal contents using the E.Z.N.A. Stool DNA Kit (D4015, Omega, Inc., Norcross, GA), according to the manufacturer's instructions. DNA concentration and purity were measured using Qubit 3.0 (Thermo Fisher Scientific, Waltham, MA) and Nanodrop One (Thermo Fisher Scientific, Waltham, MA).

Sequencing libraries were generated using NEBNext UltraTM DNA Library Prep Kit for Illumina (NEB) following the manufacturer's recommendations. Then, the established libraries were subjected to quality control (**QC**), the library was sequenced on an Illumina NovaSeq 6,000 platform (Illumina, San Diego, CA) and 150 bp paired-end reads were generated.

First, The Raw Data processing using Trimmomatic (v.0.36) was conducted to acquire the Clean Data for subsequent analysis (Chen et al., 2018). MEGAHIT (V1.0.6) was used for the de novo splicing of clean data (Peng et al., 2012). Filter the fragment shorter than 500 bp in all of Scaftigs for statistical analysis. The Scaftigs (≥500 bp) assembled from both single and mixed are all predicted the ORF by MetaGeneMark (V 3.38) (Mach et al., 2015). The CD-HIT (V 4.7) is adopted to remove redundancy and obtain the unique initial gene catalogue (Fu et al., 2012). The BBMap mapping tool was used to calculate the abundance of each unigene in each sample by comparing clean data after QC with the gene catalogue (Bushnell et al., 2014). Using DIAMOND software (v0.9.24, threshold value <=1e-5) (Buchfink et al., 2015), the unigene sequences of the non-redundant gene set were compared with the NCBI-NR species annotation database to obtain species annotation information for the unigenes and combined with the gene abundance table to obtain species composition and abundance information at each taxonomic level, and the predicted gene protein sequences were used to obtain functional annotation information by comparison with KEGG, NOG, CAZy, and other databases.

### Statistical Analysis

One-way ANOVA was used to analyze the quantity of lipid biochemical indicators and raw reads in the 3 treatment groups. performed using SPSS (v21), The results were expressed as mean ± standard error. The standards were as follows: *P* ≤ 0.05, significant difference; *P* > 0.05, nonsignificant difference. A Venn graph was plotted using the Venn diagram package of R. Histograms and heat maps were generated using GraphPad Prism (v7.0). Cluster analysis heatmaps were generated using the “Pheatmap” package in R. Principal coordinate analysis (**PCoA**) was performed using the vegan package and plotted using the “ggplot2” package in R (v0.7). Statistical analysis of similarities (**ANOSIM**) was performed using the “vegan” package (v1.17-4) in R to determine whether species differences between the groups were significant. The biomarker features in each group were screened using the linear discriminant analysis effect size (**LEfSe**) (v1.0) algorithm.

## RESULTS

### TG, T-CHO, LDL-C and HDL-C in the Liver

**Figure 1:**
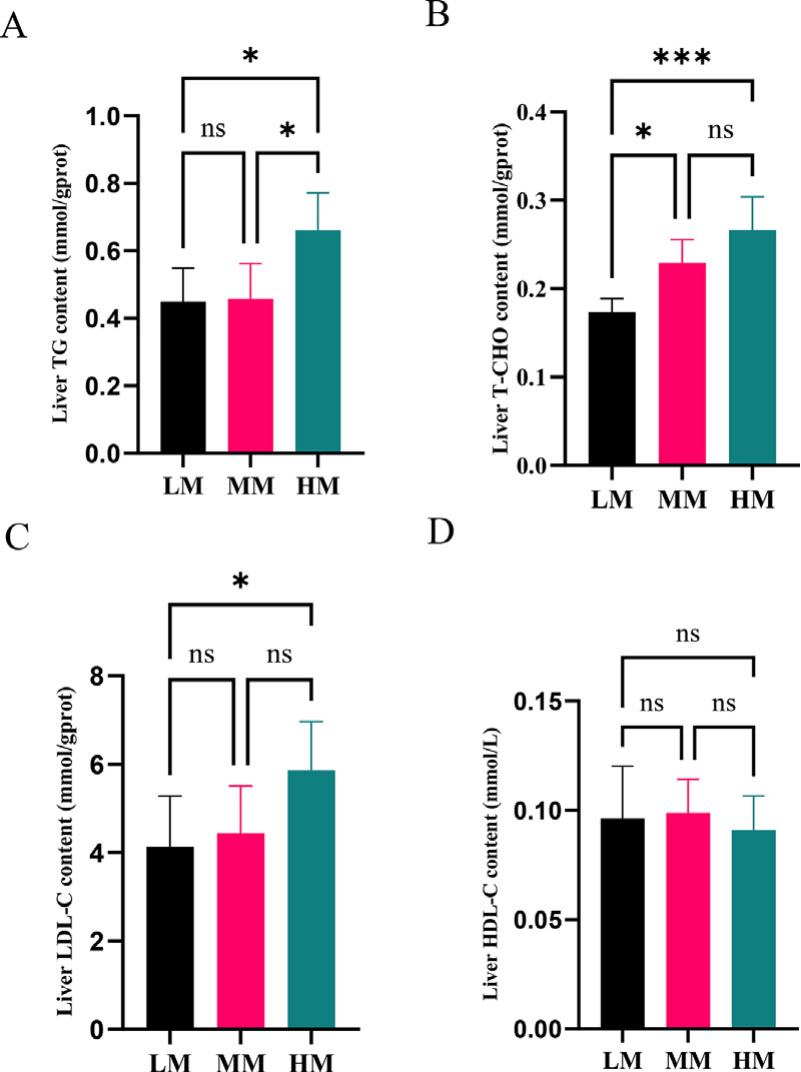
Hepatic lipid biochemical parameters of laying hens. The concentrations of TG (A), T-CHO (B), LDL-C (C) and HDL-C (D) in liver. *P ≤ 0.05, ***P ≤ 0.001, ^ns^*P* > 0.05. Abbreviations: TG, Triglyceride; T-CHO, Total cholesterol; LDL-C, low density lipoprotein cholesterol; HDL-C, high density lipoprotein cholesterol. HM, 11.56 MJ/kg metabolizable energy; MM, 11.14 MJ/kg metabolizable energy; LM, 10.72 MJ/kg metabolizable energy.

Compared with the LM group, the HM group exhibited higher TG, T-CHO, and LDL-C contents in the livers of aged laying hens (Figure 1A, B, C; *P* < 0.05), and the MM group exhibited higher T-CHO content (Figure 1B; *P* < 0.05). Compared to the MM group, the HM group exhibited a higher TG content (Figure 1A; *P* < 0.05). The content of HDL-C had no differences among the 3 groups (Figure 1D; *P* > 0.05).

### Lipid Metabolism-Related Enzymes in the Liver

**Figure 2:**
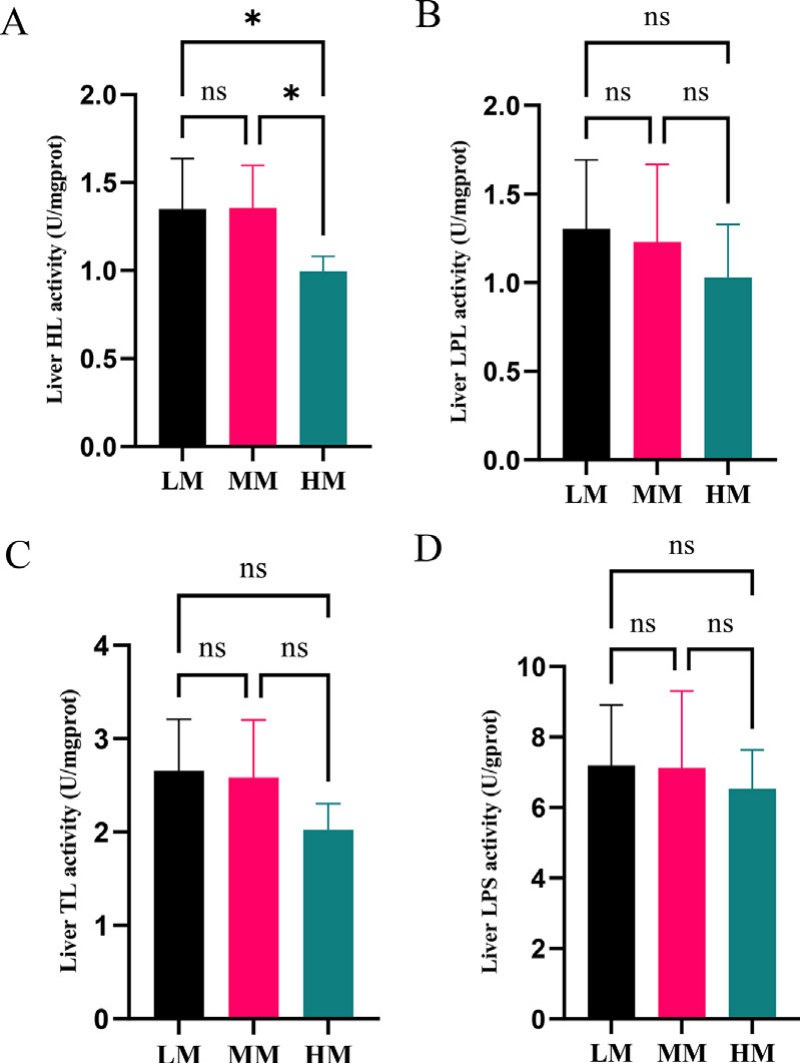
Hepatic lipid biochemical parameters of laying hens. The activity of HL (A), LPL (B), TL (C) and LPS (D) in liver. Abbreviations: HL, Hepatic Lipase; LPL, Lipoprotein Lipase; TL, Total Lipase, TL = HL + LPL; LPS, Lipase. HM, 11.56 MJ/kg metabolizable energy; MM, 11.14 MJ/kg metabolizable energy; LM, 10.72 MJ/kg metabolizable energy.

HL activity was lower in the HM group than in the LM and MM groups (Figure 2A; *P* < 0.05), whereas no significant differences were observed in the activities of the other enzymes (LPL, TL, and LPS) among the 3 groups (Figures 2B, 2C, and 2D; *P* > 0.05). There was a decreasing trend in the LPL, TL, and LPS as the energy level increased (Figures 2B, 2C, and 2D).

### Summary of the Metagenomic Datasets

A total of 1,458,978,410 raw reads and 218,847,000,000 raw bases were obtained. After filtering, 1,202,350,842 clean reads and 178,038,000,000 clean bases were obtained. 100, 99.71, 49.50, and 81.42% for clean_Q20, clean_Q30, clean_GC and Effective, respectively (Table S1).

### Microbial Composition of the Cecum

**Figure 3:**
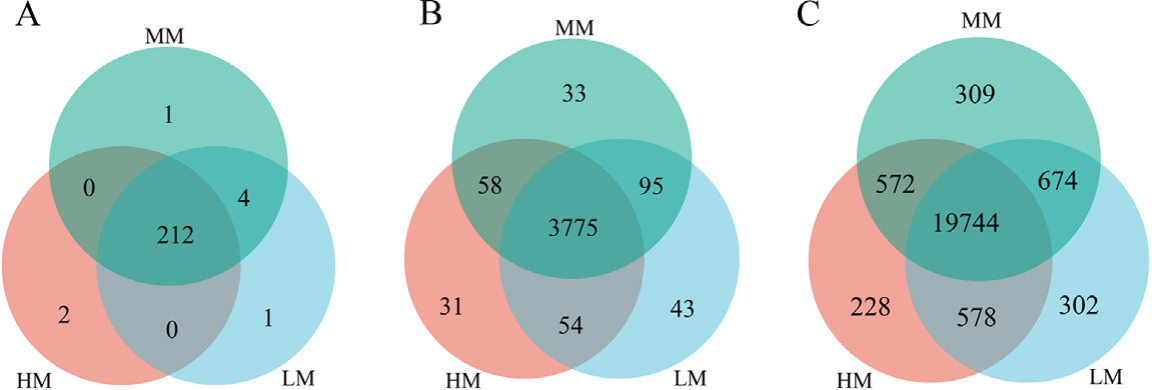
Venn plot analysis of common or endemic species numbers. Phylum(A),genus (B) and species (C) level. Abbreviations: HM, 11.56 MJ/kg metabolizable energy; MM, 11.14 MJ/kg metabolizable energy; LM, 10.72 MJ/kg metabolizable energy.

At the phylum level, 212 phyla were found in all 3 groups, with only 4 of these phyla in the LM and MM groups (Figure 3A). At the genus level, 3775 genera were found in the 3 groups, with 95 of these genera in the LM and MM groups, 58 genera in the HM and MM groups, and 54 genera in the HM and LM groups (Figure 3B). At the species level, with 19,744 species common to all 3 groups, the LM and MM groups contained 674 species, the HM and MM groups had 572 species, and the HM and LM groups contained 578 species (Figure 3C).

### Analysis of the Microbiological Composition of the Cecum

**Figure 4:**
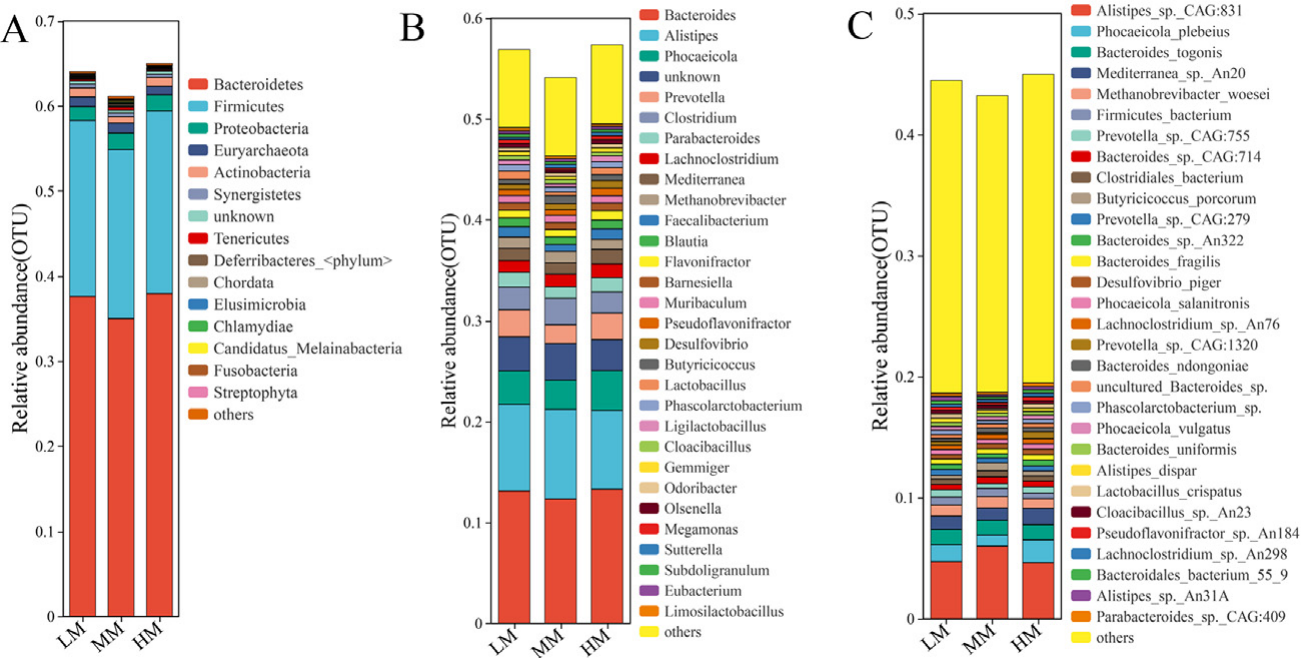
Histogram of cecal microbiota composition (A: Phylum; B: Genus; C: Specie) at different energy levels. Abbreviations: HM, 11.56 MJ/kg metabolizable energy; MM, 11.14 MJ/kg metabolizable energy; LM, 10.72 MJ/kg metabolizable energy.

Figure 4A shows that the composition of the top ten phyla was approximately equal in each treatment group, with Bacteroidetes, Firmicutes, and Proteobacteria being the 3 dominant phyla in all 3 treatment groups. The relative abundance of Proteobacteria decreased at low ME levels (Figure S1A). Figure 4B shows the composition of the gut microbiota at the genus level in each group. The dominant genera in the group were *Bacteroides, Alistipes, Phocaeicola*, “unknown”, *Prevotella, Clostridium, Parabacteroides*. Furthermore, *Parabacteroides, Lactobacillus, Phascolarctobacterium, Subdoligranulum, Limosilactobacillus* demonstrated maximum abundances at low ME levels (Figure S1B). *Desulfovibrio, Lachnoclostridium, Sutterella*, and *Butyricicoccus* showed gradual decrease in abundance with decreasing dietary ME levels and showed minimum abundances at low ME levels (Figure S1B). Figure 4C shows the composition of gut microbiota at the species level in each treatment group. The predominant species in the 3 treatment groups were *Alistipes sp. CAG:831, Phocaeicola plebeius, Bacteroides togonis, Mediterranea sp. An20, Methanobrevibacter woesei, Firmicutes bacterium, Prevotella sp. CAG:755, Bacteroides sp. CAG:714, Clostridiales bacterium, Butyricicoccus porcorum*.

### β Diversity Analysis

The PCoA results were confirmed by ANOSIM (Table 2), revealing that the difference between the HM and MM groups was significantly greater than, and the difference between the treatment groups was higher than, those within the groups (R = 0.294, *P* = 0.037). No significant differences were observed among the other groups.

**Table 2:** Analysis of inter group differences based on Anoism.

Level	dist	test	sub-condition1 vs sub-condition2	R^1^	*P-value*
Species	bray	Anosim	LM vs. MM	0.0278	0.346
Species	bray	Anosim	HM vs. MM	0.2940	0.037
Species	bray	Anosim	HM vs. LM	−0.0667	0.721

Abbreviations: HM, 11.56 MJ/kg metabolizable energy; MM, 11.14 MJ/kg metabolizable energy; LM, 10.72 MJ/kg metabolizable energy.

1 “R” is the index of ANOSIM that indicates the similarity of comparison group pairs. R-value ranged from -1 to 1, with R > 0 indicating higher intergroup differences than intragroup differences, conversely, R < 0 indicating lower intergroup differences than intragroup differences.

### LEfSe Analysis of Species

**Figure 5:**
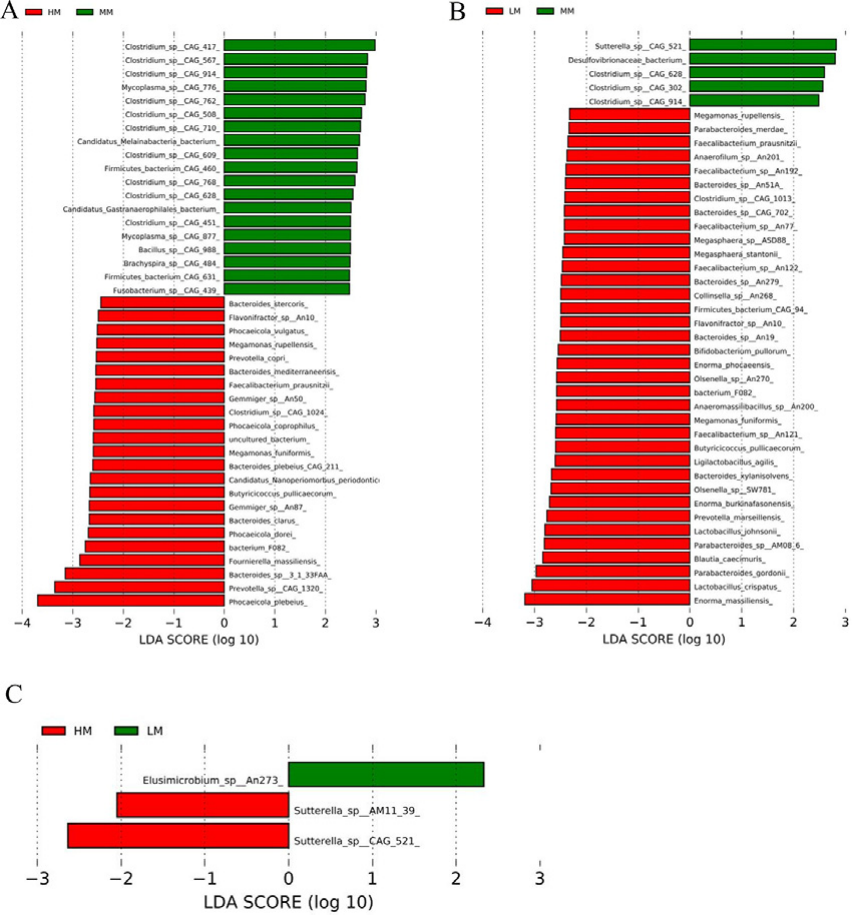
Linear discrimination analysis coupled with effect size identified the most differentially abundant taxa in the cecum microbiota (Species level). (A) HM vs. MM; (B) LM vs MM; (C) HM vs LM. Abbreviations: HM, 11.56 MJ/kg metabolizable energy; MM, 11.14 MJ/kg metabolizable energy; LM, 10.72 MJ/kg metabolizable energy.

To identify the specific bacteria that were characteristic of the 3 groups, LEfSe was used (expressed as values of linear discriminant analysis [**LDA**]) in further evaluating the differences in bacterial composition among the different dietary treatments. At the phylum level, considering the MM vs. HM treatment groups, Echinodermata and “unknown” were enriched in the HM group, while Fusobacteria, Spirochaetes, Tenericutes and Candidatus were enriched in the MM group (Figure S2A). Verrucomicrobia was enriched in the MM group relative to the LM group (Figure S2B), and the cecum chyme of the LM group was enriched in Elusimicrobia relative to the HM group (Figure S2C). At the genus level, compared to HM, MM cecum chyme samples had higher proportions of “unknown”, *Mycoplasma, Bacillus, Fusobacterium* and *Brachyspira* (Figure S3A), whereas *Elusimicrobium* was enriched in the LM group (Figure S3C). The cecum chyme of MM was more enriched in *Sutterella* relative to that of the LM group, and potentially beneficial bacteria, including *Prevotella, Faecalibacterium, Lactobacillus, Megamonas, Megasphaera, Bifidobacterium*, and *Enorma*, were enriched in the LM group compared with MM group (Figure S3B). At the species level, the 3 groups contained diverse communities of different bacterial species in the cecum. A variety of species were enriched in the HM group compared to the MM group, including *Phocaeicola plebeius, Prevotella sp. CAG: 1320, Bacteroides sp. 3_1_33FAA, Fournierella massiliensis*, and *Phocaeicola dorei, Bacteroides clarus*, as well as others (Figure 5A). In the LM treatment group, *Enorma massiliensis, Lactobacillus crispatus, Parabacteroides gordonii, Blautia caecimuris, Lactobacillus johnsonii, Prevotella marseillensis*, and others were enriched compared to the MM group (Figure 5B). In addition, the relative abundances of *Desulfovibrionaceae bacterium* and *Sutterella sp. CAG: 521*, and others were significantly decreased in the LM group (Figure 5B). Comparing LM with HM revealed that the species with higher numbers in the HM group was *Sutterella spp.*, while *Elusimicrobium sp*. was enriched in the LM group (Figure 5C).

### KEGG, NOG, and CAZy Analyses

**Figure 6:**
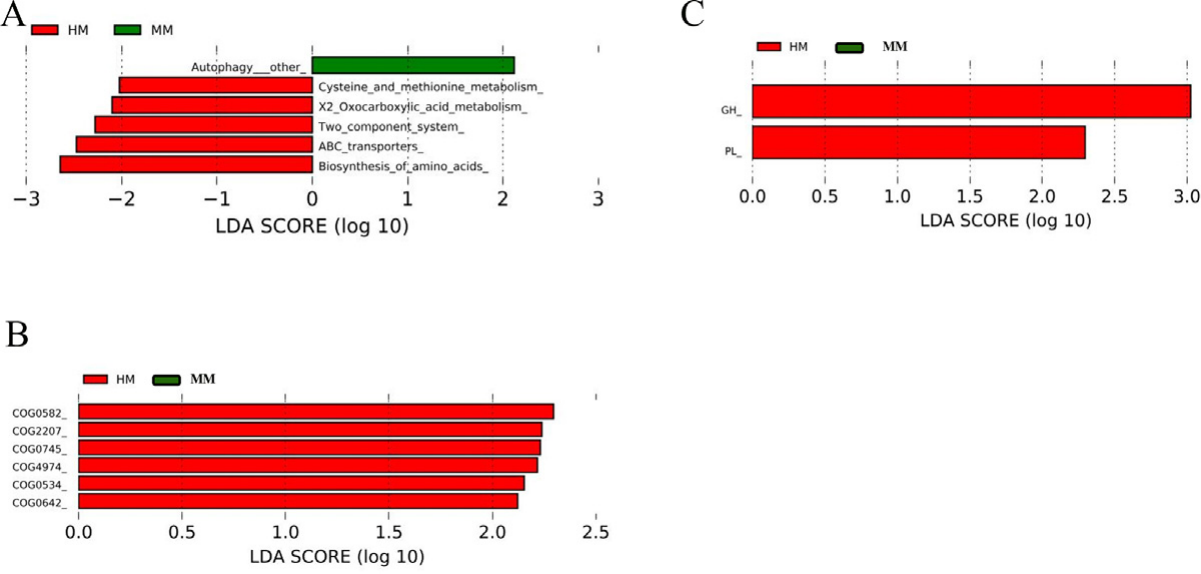
Linear discrimination analysis coupled with effect size (LEfSe) identified the most differentially abundant functions. (A) HM vs. MM, KEGG, L3; (B) HM vs. MM, NOG, L2; (C) HM vs. MM, CAZy, L1. No significant difference for other groups. Abbreviations: HM, 11.56 MJ/kg metabolizable energy; MM, 11.14 MJ/kg metabolizable energy; LM, 10.72 MJ/kg metabolizable energy.

KEGG pathway analysis indicated that the biosynthesis of amino acids, ABC transporters, X2_Oxocarboxylic acid metabolism and Cysteine and methionine metabolism were enriched in the HM group compared to the MM group, whereas “autophagy other” was enriched in the MM group (Figure 6A).

The NOG pathway analysis demonstrated that integrase/recombinase includes phage integrase (COG0582), AraC-type DNA-binding domain and AraC-containing proteins [Transcription] (COG2207), DNA-binding response regulator, OmpR family, contains REC and winged-helix (**wHTH**) domain (COG0745), Site-specific recombinase XerD [Replication, recombination and repair] (COG4974), Na+-driven multidrug efflux pump [Defense mechanisms] (COG0534), Signal transduction histidine kinase [Signal transduction mechanisms] (COG0642) were enriched in the HM group compared to the MM group (Figure 6B).

Metagenomic data were aligned using the CAZy database. The results showed that glycoside hydrolases and Polysaccharide Lyases were significantly more abundant in the HM group compared to the MM group (Figure 6C).

## DISCUSSION

After high-intensity production, laying hens enter the aged laying period when their production performance, egg quality, and body metabolism tend to decline, and the laying rate is low (Yao et al., 2023). The liver is an important metabolic organ in animals and plays a major role in lipid metabolism (Rui et al., 2014). The metabolic capacity of the liver decreases dramatically as laying hens enter the aging stage of the egg-laying period (Gu et al., 2021). Dietary energy levels affect various nutritional responses (Kim et al., 2022). In laying hens, excessive fat intake can increase the burden of lipid metabolism in the liver, leading to several metabolic diseases (Zhang et al., 2023). Multiple liver indicators, including TG, T-CHO, LDL-C, and HDL-C, are strongly associated with metabolic syndrome. TG is a form of lipid storage in hepatocytes, and its abnormal secretion often leads to excessive lipid storage in the liver (De la Rosa Rodriguez et al., 2021). LDL-C transports lipids from the liver to the blood vessels, causing disease, and its high levels are associated with abnormal lipid metabolism (Natesan et al., 2021). HDL-C transports lipids from the blood vessels to the liver and carries lipids from the blood vessels to the liver, where they are broken down, protecting against heart disease and other diseases of the cardiovascular system (Natesan et al., 2021). One previous study indicated that reducing energy intake will reduce fat deposition and may be associated with food restriction (Duregon et al., 2021). It can be assumed that the metabolism of laying hens favors continuous utilization of energy to meet egg production requirements rather than storage during the periods of energy reduction (Murugesan et al., 2013). In the present study, the data indicated that the concentrations of TG, T-CHO, and LDL-C in the liver tended to increase. Specifically, their concentrations significantly increased in the HM group, and significantly decreased when ME levels in the diet were reduced, compared to those of the MM group. Consistent with our findings, a similar result was observed by Xia et al. (2019), who demonstrated that disregarding the CP level, in a diet with 2,500 kcal/kg ME, lipid metabolism in the liver increased and abdominal fat decreased (Xia et al., 2019). Another report revealed that decreasing energy density reduced protein and lipid gain (Kim et al., 2022).

In the liver, several enzymes, including HL, LPL, TL, and LPS, play important roles in lipid metabolism (Feingold et al., 2022). Our results indicate that as ME levels increased, the activity of HL significantly decreased, and the activities of LPL, TL, and LPS showed decreasing trends, but these were not significant. As a lipase mainly secreted from the liver, hepatic lipase (**HL**) is closed associated with obesity (Quiroga et al., 2012). Accordingly, there have been reports indicating that HL promoted triglyceride-rich lipoprotein hydrolysis and adipose FFA uptake (Cedó et al., 2017). In summary, we speculate that reducing energy levels and fixing feeding rates may reduce the ability of laying hens to accumulate liver fat.

Microbial colonization of the host is highly susceptible to dietary influences, and one of the guarantees of gut health in laying hens is the presence of a normal and stable flora (Velagapudi et al., 2010; Kers et al., 2018). Microorganisms in the gut are often involved in host-related metabolic activities and alter the gut morphology to influence nutrient digestion, absorption, and feed conversion, thereby regulating animal growth and metabolism (Pan et al., 2014). The cecum is the main functional part of the distal intestine and has a rich flora that plays an important role in avian metabolism (Polansky et al., 2015). This includes the ability to prevent pathogen colonization, as well as to detoxify pollutants, recycle nitrogen, and absorb additional nutrients (Yan et al., 2017). The major phyla in the avian gut microbiota are Firmicutes, Bacteroidetes, Fusobacteria, Actinobacteria, and Proteobacteria (Khan et al., 2020). In the present study, we found that Bacteroidetes, Firmicutes, and Proteobacteria were the dominant phyla in the 3 groups of gut microbiota, consistent with the results of Oakley et al. (2014). Firmicutes primarily help the host absorb energy and tend to cause weight gain (Zhang et al., 2020). Bacteroidetes are involved in carbohydrate metabolism, breaking down sugars to produce volatile fatty acids, which are then absorbed and utilized by the gut (Johnson et al., 2015). Proteobacteria is the largest phylum of bacteria and is predominantly pathogenic (Rizzatti et al., 2017). The abundance of Proteobacteria in animals is positively correlated with the incidence of certain diseases, often associated with obesity and diabetes, and metabolic disorders occur when their levels increase (Moon et al., 2018). In this study, decreasing the dietary ME levels decreased the abundance of Proteobacteria. A previous experiment in broiler chickens also confirmed that an increase in Proteobacteria abundance caused gut microbiota dysbiosis and hepatic lipid metabolism disorders in chickens (Kong et al., 2020). Therefore, to a certain extent, homeostasis of the gut microbiota can be maintained by reducing dietary ME levels. This study compared differential phyla between the 2 groups using LEfSe analysis, finding that Elusimicrobia was enriched in the LM group compared to the HM group. Gut-associated Elusimicrobia rely on fermentation. Interestingly, they can produce and use H^++^2 (Méheust et al., 2020). Thus, it can be hypothesized that when the energy content of the diet is reduced, laying hens may be able to provide energy through fermentative metabolism.

*Prevotella* is a producer of short-chain fatty acids that supply energy to intestinal cells of the intestine (Lin et al., 2022). Research has found that *Prevotella* plays a role in improving glucose metabolism, potentially by promoting increased glycogen storage (Kovatcheva et al., 2015). However, in pig intestines, the accumulation of *Prevotella* has been reported to cause ecological disturbances (Zhao et al., 2022). Therefore, the effect of increased *Prevotella* abundance in the cecal microbiology of low-energy laying hens requires further investigation. *Lactobacillus* and *Bifidobacterium* are important probiotics in the intestinal tract (Presti et al., 2015). *Lactobacillus* express tryptophanase, which is the most important commensal protein that metabolizes tryptophan (McCarville et al., 2020). A higher abundance of *Lactobacillus* in the cecal contents of hens was found to improve feed conversion efficiency and enrich amino acid and carbohydrate-related metabolic pathways (Yan et al., 2017). *Bifidobacterium* may have beneficial health effects by regulating microbial homeostasis, reducing intestinal lipopolysaccharide levels, and improving mucosal barrier function (Kim et al., 2023). One study found that the abundance of *Bifidobacterium* was reduced in mice fed a high-energy diet (Cani et al., 2007). *Bifidobacterium* can synthesize beneficial VB for animals by participating in their metabolism, convert minerals into ions that can be easily absorbed by the animal to improve utilization, and have fat reducing and glycolipid regulating effects (LeBlanc et al., 2013). It has been reported that *Fecalibacterium* is a next-generation probiotic or live biotherapeutic product, and the relative presence of this genus is considered to reflect intestinal health status (De Filippis et al., 2022). *Fecalibacterium* is frequently present at reduced levels in individuals with gastrointestinal diseases or disorders (Martín et al., 2023). Studies have shown that *Sutterella* is positively associated with obesity and contributes to gut dysbiosis (Zhang et al., 2020; Zhao et al., 2022). In the present study, compared to the MM group, *Prevotella, Lactobacillus, Fecalibacterium, Bifidobacterium* were significantly enriched in the LM group, but *Sutterella* were significantly decreased in the LM group. In addition, compared to the HM group, we found *Elusimicrobium* were significantly enriched in LM group. Reportedly, *Elusimicrobium* was the potential dominant bacteria that developed modifications of bile acid, amino acid, and fatty acid (Wang et al., 2022). These results suggest that reduced ME leads to lower fat accumulation owing to changes in gut microorganisms.

Intrigued by the data at the species level, we conducted comparative analyses at the species level to facilitate the derivation of particular species that varied at different energy levels. We observed that *Phocaeicola plebeius, Bacteroides sp. 3_1_33FAA, Phocaeicola dorei*, and *Bacteroides Clarus* were enriched in the HM group compared to the MM group. *Phocaeicola plebeius* has been reported to survive better in a carbohydrate-rich gut environment, and might promote the expression of genes encoding α-galactosidase and α-glucosidase (Li et al., 2022). As supportive evidence, glycoside hydrolases and Polysaccharide Lyases were enriched in CAZy profiles of the HM group compared to those of the MM group. The increased amount of substrate that must be degraded when energy levels are elevated may lead to the enrichment of degrading bacteria and enzymes in the gut. Reportedly, *Bacteroides sp. 3_1_33FAA, Phocaeicola dorei*, and *Bacteroides Clarus* were enriched in patients with diabetes (Wu et al., 2020; Debédat et al., 2022; Lei et al., 2023).

In the present study, we found that *Lactobacillus crispatus, Parabacteroides gordonii, Blautia caecimuris, Parabacteroides sp., Lactobacillus johnsonii*, and others were significantly enriched in the LM group compared to the MM group, but *Desulfovibrionaceae bacterium, Sutterella sp. CAG: 521*, and others were significantly decreased. These microorganisms represent potential probiotics, and the maintenance of intestinal and hepatic metabolic homeostasis at low energy levels may be related to their enrichment (Arora et al., 2013). *Lactobacillus crispatus* has multiple physiological functions, including maintaining microbial balance, improve digestive function, and enhance immunity (Nouri et al., 2010). It can be assumed that the enrichment of *Lactobacillus_crispatus* at low energy levels stimulates the potential for glycogen utilization by the organism, thus providing the required energy. *Blautia caecimuris*, as the dominant bacteria in the intestinal microbiota, has a significant correlation with the physiological dysfunction of the host, such as obesity, diabetes, cancer and various inflammatory diseases (Luu et al., 2023). *Parabacteroides spp.* may be viewed as potential next-generation probiotic candidates due to their protective effects on inflammation and obesity in mice (Cui et al., 2022). In addition, it has been reported that *Lactobacillus_johnsonii* ameliorates not only intestinal health, but also extra-intestinal health, including hepatic health (Bereswill et al., 2017).

## CONCLUSIONS

In summary, appropriately reduced energy levels maintained the lipid metabolic capacity of the liver in aged laying hens. In addition, reduced energy levels improved gut homeostasis and increased the levels of beneficial bacteria, including *Lactobacillus crispatus, Parabacteroides gordonii*, and *Lactobacillus johnsonii*, as well as decreased the abundance of Proteobacteria. Increased energy levels interfered with liver metabolism and increased the abundance of Sutterella in the cecum, resulting in fatty deposits.

## Appendix Supplementary materials

Image, application 1

## Footnotes

Supplementary material associated with this article can be found in the online version at doi:10.1016/j.psj.2024.103855.
