# Supplementary material for: Effects of dietary metabolizable energy level on hepatic lipid metabolism and cecal microbiota in aged laying hens
Source: Poult Sci. 2024 May 15;103(7):103855. doi: 10.1016/j.psj.2024.103855 (PMC11153248; doi:10.1016/j.psj.2024.103855)
Supplement: Supplementary file 1 [file mmc1.docx]

#### **Supporting information for**

**Effects of dietary metabolizable energy level on hepatic lipid metabolism and cecal microbiota in aged laying hens**

**Short title：EFFECT OF ENERGY LEVEL ON AGED LAYING HENS**

Anjian Li^1,^ **^†^**, Hong Hu^1,^ **^†^**, Ying Huang^1^, Fuyan Yang^1^, Qianhui Mi^1^, Liqiang Jin^1^, Hongli Liu^1^, Qiang Zhang^2^, Hongbin Pan*

**Affiliation and address**

^1^Yunnan Provincial Key Laboratory of Animal Nutrition and Feed Science, Faculty of Animal Science and Technology, Yunnan Agricultural University, Kunming, 650201, China

^2^ WOD Poultry Research Institute, Beijing, 100193, China

**Note: ^†^** These authors contributed equally to the experimentations

**Scientific section: Nutrition and Microbiota**

**The authors have declared that no competing interests exist.**

***Corresponding authors：ynsdyz@163.com**

Table S1 Sequencing quality statistics per sample

| Sample | Raw reads | Raw bases | Clean reads | Clean bases | Clean_Q20(%) | Clean_Q30(%) | Clean_GC(%) | Effective(%) |
| --- | --- | --- | --- | --- | --- | --- | --- | --- |
| LM1 | 79183364 | 11877504600 | 65403254 | 9679547953 | 100 | 99.72 | 49.0 | 81.49 |
| LM2 | 85116322 | 12767448300 | 70205886 | 10407125404 | 100 | 99.74 | 49.0 | 81.51 |
| LM3 | 88934744 | 13340211600 | 72932486 | 10809496971 | 100 | 99.72 | 49.0 | 81.03 |
| LM4 | 99267634 | 14890145100 | 80560884 | 11935915600 | 100 | 99.72 | 50.0 | 80.16 |
| LM5 | 92220028 | 13833004200 | 75095036 | 11130547733 | 100 | 99.74 | 51.5 | 80.46 |
| LM6 | 79593228 | 11938984200 | 66080746 | 9786891728 | 100 | 99.70 | 49.0 | 81.97 |
| MM1 | 85988032 | 12898204800 | 71341414 | 10554341974 | 100 | 99.69 | 49.0 | 81.83 |
| MM2 | 89563582 | 13434537300 | 72409330 | 10733112948 | 100 | 99.74 | 50.0 | 79.89 |
| MM3 | 72622524 | 10893378600 | 60339224 | 8882489490 | 100 | 99.56 | 49.0 | 81.54 |
| MM4 | 72116634 | 10817495100 | 60667162 | 8988502465 | 100 | 99.73 | 49.0 | 83.09 |
| MM5 | 68077286 | 10211592900 | 55093912 | 8156350629 | 100 | 99.72 | 49.0 | 79.87 |
| MM6 | 69127134 | 10369070100 | 57449520 | 8506972876 | 100 | 99.70 | 49.0 | 82.04 |
| HM1 | 84276838 | 12641525700 | 69380458 | 10260603658 | 100 | 99.67 | 51.0 | 81.17 |
| HM2 | 87868734 | 13180310100 | 72373402 | 10714746470 | 100 | 99.72 | 51.0 | 81.29 |
| HM3 | 81740470 | 12261070500 | 68388402 | 10114485443 | 100 | 99.68 | 50.0 | 82.49 |
| HM4 | 89280028 | 13392004200 | 72549156 | 10759311643 | 100 | 99.75 | 49.0 | 80.34 |
| HM5 | 66496220 | 9974433000 | 55737628 | 8266260581 | 100 | 99.75 | 49.0 | 82.87 |
| HM6 | 67505608 | 10125841200 | 56342942 | 8351773529 | 100 | 99.74 | 49.0 | 82.48 |

Abbreviations: LM1-LM6: 10.72 MJ/kg metabolizable energy; MM1-MM6: 11.14 MJ/kg metabolizable energy; HM1-HM6: 11.56 MJ/kg metabolizable energy.


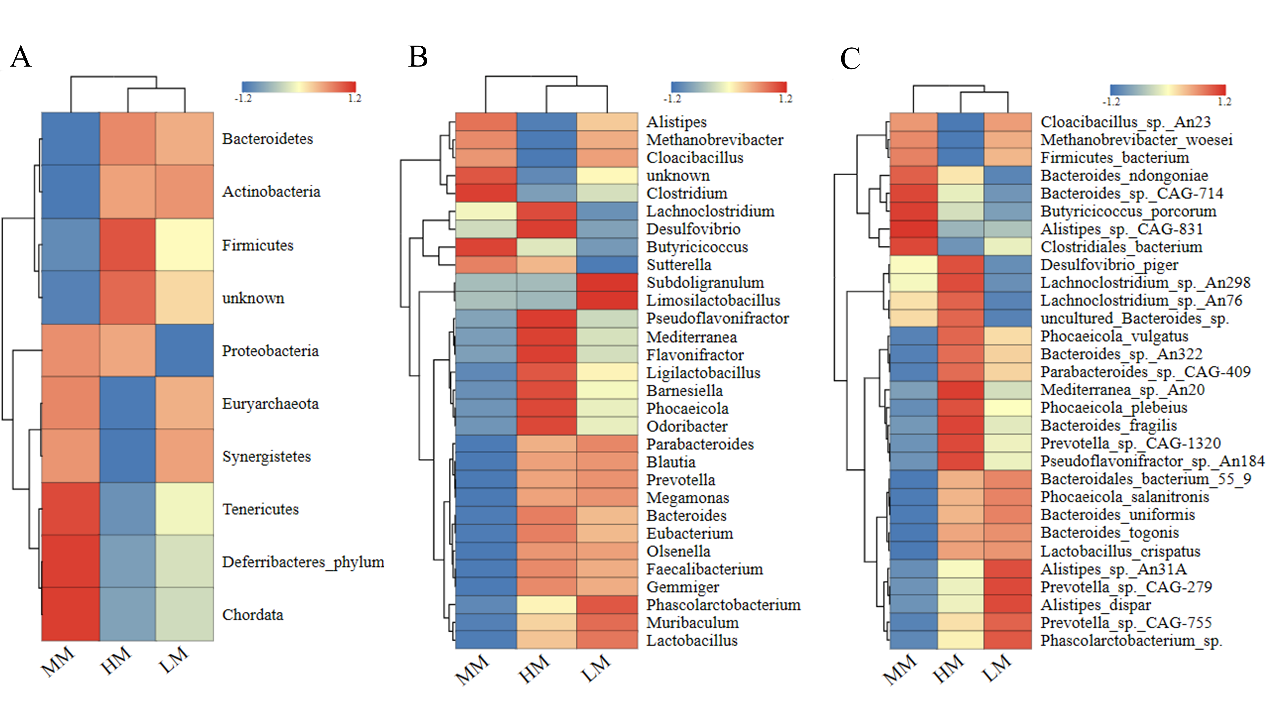


**Figure S1.** Cluster heatmap of relative abundance of gut microbiota at phylum and genus levels. (A) cluster heat map at the phylum level, (B) cluster heat map at the genus level. (C) cluster heat map at the species level. Red represents high abundance, blue represents low abundance, and white represents intermediate abundance. Abbreviations: HM, 11.56 MJ/kg metabolizable energy; MM, 11.14 MJ/kg metabolizable energy; LM, 10.72 MJ/kg metabolizable energy.


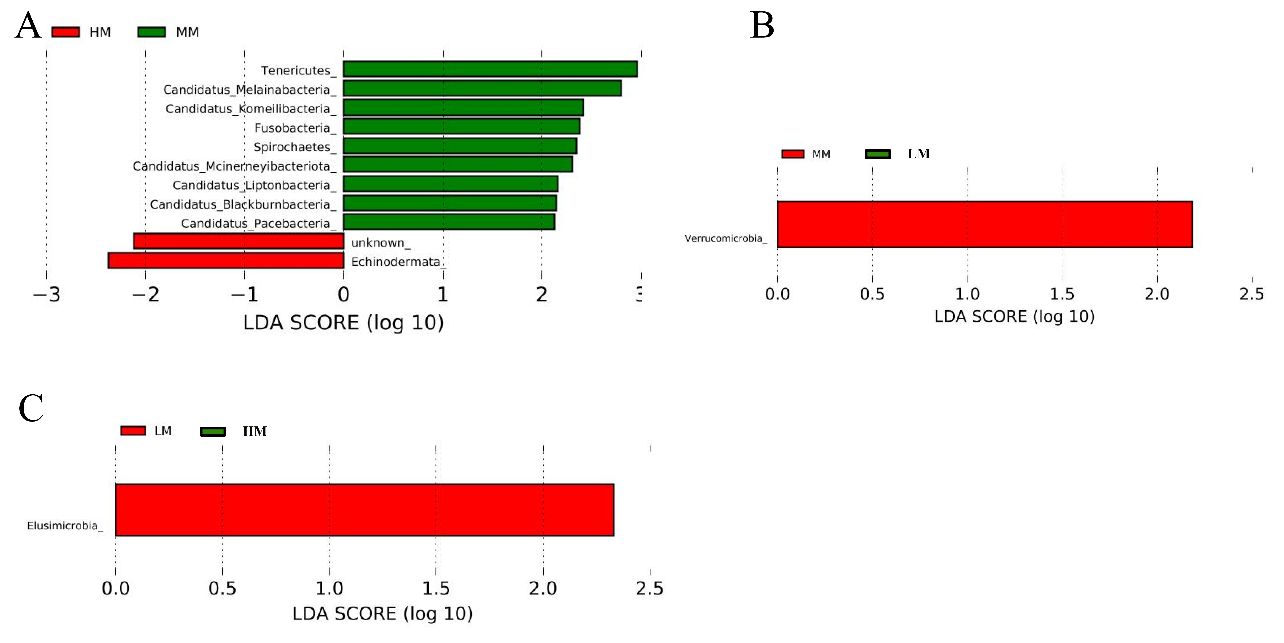


**Figure S2.** Linear discrimination analysis coupled with effect size identified the most differentially abundant taxa in the cecum microbiota (Phylum level). (A) HM vs. MM; (B) LM vs. MM; (C) LM vs. HM. Abbreviations: HM, 11.56 MJ/kg metabolizable energy; MM, 11.14 MJ/kg metabolizable energy; LM, 10.72 MJ/kg metabolizable energy.


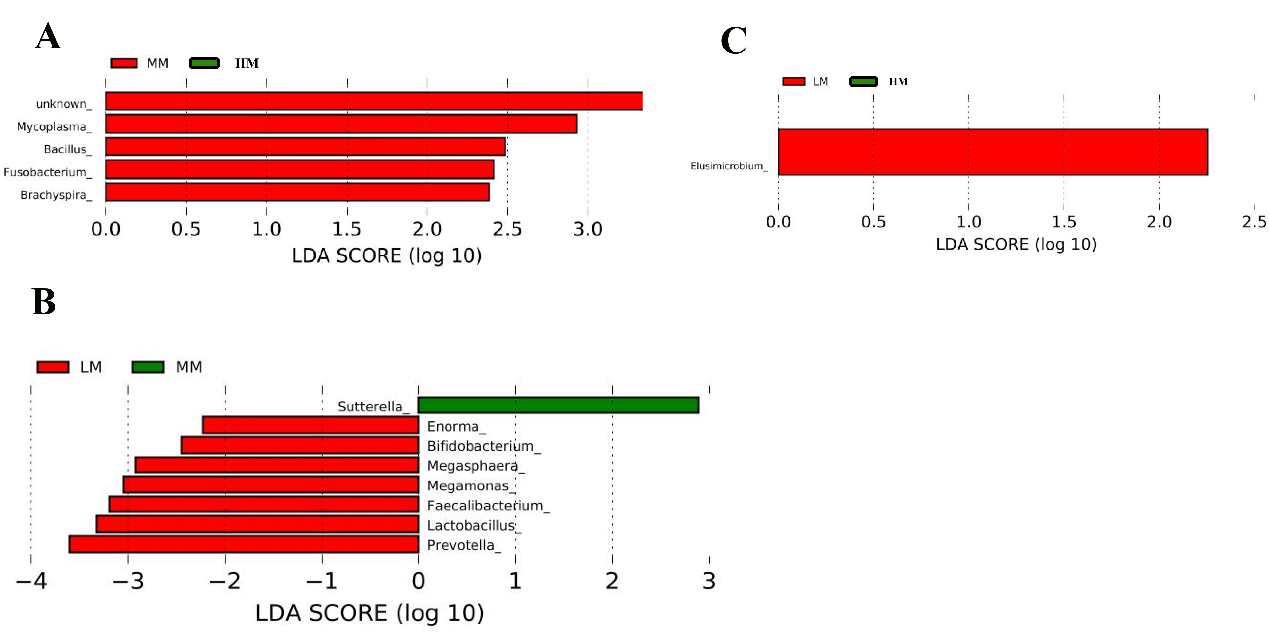


**Figure S3.** Linear discrimination analysis coupled with effect size identified the most differentially abundant taxa in the cecum microbiota (Genus level). (A) HM vs. MM; (B) LM vs. MM; (C) HM vs. LM. Abbreviations: HM, 11.56 MJ/kg metabolizable energy; MM, 11.14 MJ/kg metabolizable energy; LM, 10.72 MJ/kg metabolizable energy.
